# Medicinal plant knowledge in Caribbean Basin: a comparative study of Afrocaribbean, Amerindian and Mestizo communities

**DOI:** 10.1186/s13002-015-0008-4

**Published:** 2015-02-25

**Authors:** Wendy Torres-Avilez, Martha Méndez-González, Rafael Durán-García, Isabelle Boulogne, Lionel Germosén-Robineau

**Affiliations:** Laboratory of Applied and Theoretical Ethnobiology (LEA), Federal Rural University of Pernambuco, Av. Dom Manoel de Medeiros s/n, Dois Irmãos, Recife, Pernambuco, CEP 52171-900 Brazil; Centro de Investigación Científica de Yucatán A.C. Unidad de Recursos Naturales, TRAMIL (Program of Applied Research to Popular Medicine in the Caribbean), Calle 43 No. 130, Colonia Chuburná de Hidalgo, Mérida, Yucatán, CP 97200 México; Université des Antilles et de la Guyane, UFR Sciences Exactes et Naturelles, Laboratoire de Biologie et de Physiologie végétales, TRAMIL, F-97157 Pointe-à-Pitre Cedex, Guadeloupe, France

**Keywords:** Medicinal flora, Caribbean Basin, Cultural intermixing, Migration, Hybridization

## Abstract

**Background:**

The Caribbean Basin has complex biogeographical and cultural histories that have shaped its highly diverse botanical and cultural landscapes. As a result, the current ethnic composition of the Basin is a heterogeneous mixture including Amerindian, Afrocaribbean and a wide range of Mestizo populations. A comparison was done of the medicinal plant repertoires used by these groups to identify the proportion of native species they include and any differences between the groups.

**Methods:**

The TRAMIL program has involved application of ethnopharmacological surveys to gather data on the medicinal plants used for primary care in 55 locations the Caribbean Basin. Three Afrocaribbean, three Amerindian and three Mestizo communities were selected taking in account the Ethnic prevalence. Differences in native and exotic plant used by groups and between groups were done using contingency tables. Identification of differences in the numbers of native and exotic plants used within each group was done with a one sample Z -test for proportions.

Similarity in medicinal species use was estimated using the Sørensen Similarity Index. Species use value (UV) was estimated and a principal components analysis (PCA) run to determine differences between groups.

**Results:**

The 1,753 plant records generated from the surveys of the nine communities included in the analysis covered 389 species from 300 genera and 98 families. The studied groups used different numbers of native and exotic species: Afrocaribbean (99 natives, 49 exotics); Amerindian (201 natives, 46 exotics); and Mestizo (63 natives, 44 exotics). The proportion of natives to exotics was significantly different in between the Afrocaribbean and Amerindian communities, and between the Amerindian and Mestizo communities, but not between the Afrocaribbean and Mestizo communities. In the PCA, the groups were disparate in terms of the use value they assigned to the medicinal species; these were determined according to species with high use value and those used exclusively be a particular group

**Conclusions:**

Although migration, cultural intermixing and a consequent hybridization of medicinal plant knowledge have occurred in the Caribbean Basin, the results highlight differences between the three studied groups in terms of the medicinal plant repertoire they employ for primary health care.

## Background

Floral diversity is distinctive in the Caribbean Basin. The region encompasses portions of North, Central and South America, as well as the Caribbean islands, some of which exhibit different degrees of isolation [1]. Regional geological and biogeographic events, in association with its geographical complexity, have promoted diversity on many fronts. In tandem with the Basin’s social history during the last five hundred years, these biogeographic events have contributed to shaping its biological diversity, with a unique mix of native and exotic species [2,3].

When European contact began in the Basin in the late 15^th^ Century, a process was begun of severe transformations in ecosystems, natural resources, human groups and cultural components [4]. Significant changes in biodiversity were initiated; for example, deforestation to make way for crops such as sugar cane, banana, tobacco and coffee, active exploitation of native plants and animals and introduction of many exotic species [5-8].

The indigenous peoples of the Caribbean islands were almost totally decimated and replaced by slaves, largely from Africa and Asia, who were used as labor in the developing cultivation and extraction systems. In the islands, slaves mainly mixed with Europeans [4,9,10], but along the Basin’s continental margins both slaves and Europeans mixed with surviving indigenous populations [9,10]. As a result, the current ethnic composition of the Basin is a heterogeneous mixture including Amerindian, Afrocaribbean and a wide range of Mestizo populations [10-13].

The cultural change initiated with European contact influenced local knowledge of pharmacopeias since the surviving indigenous populations were forced to adopt new cultural practices, and enter into new economic and political processes [14,15]. Human migration and the cultural and racial intermixture that it assumes [16-18], clearly contributed to an exchange of medicinal plant species, allowing the introduction of new species and the development of new uses for existing species. Native pharmacopeias consequently diversified.

Since 1982, the TRAMIL program (Program of Applied Research to Popular Medicine in the Caribbean) has been documenting medicinal plants and their uses throughout the countries of the Caribbean Basin [19] and taking into account that medicinal resource selection and use are influenced by culture (indigenous and/or introduced), regional resource availability and time of residence [20-23], the present study objective was to determine if indigenous populations, given their experience exploiting native floral diversity, use a larger number of native plants than exotics, and if differences exist between Caribbean Basin Afrocaribbean, Amerindian and Mestizo communities in terms of their knowledge and use of medicinal plant resources.

## Methods

### Study area

The Caribbean Basin consists of the eastern Yucatan Peninsula, Central America, northern South America, the Bahamas and the Greater and Lesser Antilles [1]. Its geology is complex, with the North American, South American and Caribbean tectonic plates all crossing the region. Floral diversity is extremely high, with high rates of endemism. Hotspots with particularly high rates of endemic species include the islands of Cuba (53%) and Hispaniola (30%) [1,24]. Myers and his collaborators [24] estimate floral species richness in South Florida and the Caribbean islands to be 12,000 species, including endemic species representing 2.3% of all vascular plants on the planet (Table 1).

**Table 1:** **Numbers of vascular plant species, estimated number of medicinal plants and percentages of endemism for all the surveyed communities**

**Study area**	**Number of vascular plant species**	**Number of medicinal plants**	**% of endemism**	**References**
Belize	4423	64	19	[25,26]
Jamaica	2746	116	31	[1,27]
Haiti	4685	161	30	[1,28]
Mexico	30 000	5000	52	[29,25]
Nicaragua	5796-7000	229	19	[25,30]
Panama	8500-9000	265	19	[25,31]
Cuba	6015	101	53	[1,32]
Puerto Rico	2128	172	10	[1,33]
Dominican Republic	4685	200	30	[1,34]

### Data collection

The data analyzed here was collected as part of TRAMIL over a thirty-two year period (1982–2014) in 55 locations in 29 countries and territories in the Basin. Data were collected via ethnopharmacological surveys based on the health problems most affecting each surveyed community. The list of health problems was elaborated by a local multidisciplinary group (nurse, social worker, mothers, traditional healer, etc.) in order to adapt it to the realities of the country. A random selection was made of 10% of the households in each community, and interviews held with the person responsible for household member health, usually the mother. Information was gathered on the plant species used in treatment of each disorder and preparation methods. The medicinal plants recorded in the interviews were identified by specialists from each country’s national plant collection, and specimens deposited in each collection. Specimens of each collected plant were also deposited in the Santo Domingo Botanical Garden Herbarium in the Dominican Republic [35].

From the total sample of communities surveyed by TRAMIL, three Afrocaribbean, three Amerindian and three Mestizo communities were selected taking in account the Ethnic prevalence; besides, the communities selected are recognized themselves as Afrocaribbean, Amerindian and Mestizo (Table 2). Afrocaribbean communities were those in which most inhabitants were descendants of the African diaspora resulting from the slave trade between the 16^th^ and 19^th^ centuries [36]. One group typical of these communities is the Garifuna, found along the coasts of Belize, Guatemala, Honduras and Nicaragua [12,30]. Amerindian communities were those in which inhabitants had preserved the language, social and cultural practices of their places of origin, despite sociocultural mixture [37]. Mestizo communities were those in which inhabitants had neither substantial Afrocaribbean nor indigenous influences, and manifested the traits of other migrant cultures, with a markedly Western lifestyle [11,38].

**Table 2:** **Selected communities in each human group in the Caribbean Basin**

**Surveyed communities**	**Number of informants**	**Ethnic prevalence**	**Surveyed communities**	**Number of informants**	**Ethnic prevalence**	**Surveyed communities**	**Number of informants**	**Ethnic prevalence**
Belize (Caribbean Coast)	200	**Afro Caribbeans**: prevalence of the African diaspora’s descendants	México (Felipe Carrillo Puerto)	252	**Amerindians**: prevalence of pre-Columbian inhabitants’ descendants	Cuba (Trinidad)	127	**Mestizos:** prevalence of mixed race persons (descendants with a mix of European, African and Amerindian characteristics)
Jamaica	270		Nicaragua (Bosawás)	139		Puerto Rico (Eastern Region)	351	
Haití (La Chapelle)	200		Panamá (Bugle)	197		República Dominicana (Zambrana- jacobo-claro)	95	

### Database

Data collected from the surveys was used to build a database (Microsoft Excel 2010^©^) divided into three sections: taxonomy; geography; and ethnobotany. Accuracy of data in the taxonomic section was confirmed by checking the main TRAMIL specimen collection in the JBSD, the herbarium at the Yucatan Center for Scientific Research (CICY), and the TROPICOS database of the Missouri Botanical Garden. Recorded health disorders were classified into fourteen categories according to the World Health Organization (WHO) international classification of diseases [39]. Species distribution was divided into native and exotic plants. Native species were those distributed naturally throughout the Caribbean Basin, while exotics were those for which natural distribution does not include the Basin. Distribution was determined by consulting fourteen national and regional floras, including those for Guatemala, the Bahamas archipelago, Cuba, Hispaniola, Mesoamerica and Nicaragua. In addition, the GRIN (US Department of Agriculture) [40] and TROPICOS (Missouri Botanical Garden) databases were consulted.

### Data analysis

Comparison among ethnic groups was done by first counting the number of plant families and species with medicinal uses in each group and then counting the number of species in each use category. Differences in native and exotic plant used by groups were done using a 2×3 contingency table; 2×2 contingency tables were done to detect differences between groups. Identification of differences in the numbers of native and exotic plants used within each group was done with a one sample z-test for proportions.

Intergroup similarities in use of a plant resource were estimated with the Sørensen Similarity Index [41]: SSI = (2c/A + B) × 100; where c = is the sum of the number of species held in common between groups, A = is the number of species for group 1 and B = is the number of species for group 2.

Use value (UV) was calculated with the formula UV = ∑U /n proposed by Phillips and Gentry [42] and modified by Rossato and his collaborators [43] to determine the species more used therapeutically in each group. A non-parametric Kruskal-Wallis test was applied to calculate the mean of a species’ use value between groups. Statistical analyses were run with the BioEstat 5.3^©^ program [44].

A principal components analysis (PCA) was run to determine the degree of group clustering based on the use value of medicinal species used by each group. This analysis was done with the MVSP 3.1^©^ program [45].

## Results and discussion

### Plant species used for the Afrocaribbean, Amerindian and Mestizo communieties

The 1,753 plant records generated from the surveys of the nine communities included in the analysis covered 389 species from 300 genera and 98 families. Among the species total, 247 were used in the Amerindian communities, 148 in the Afrocaribbean communities and 107 in the Mestizo communities. The surveyed Amerindian communities used 78 plant families, the Afrocaribbeans used 64 and the Mestizos used 56. Four botanical families accounted for the highest number of species in all three groups: Fabaceae, Asteraceae, Lamiaceae and Euphorbiaceae. These families are well represented in the Caribbean flora and coincides with their high diversity worldwide [46]; indeed; the same results have been reported for the first three families in countries as varied as China, Peru, Ethiopia, Malaysia, Italy and Argentina [47-52]. Moerman [53] states that the high richness of species with medicinal applications in some plant families is determined by the chemical components they contain, and that specific families can be more effective in treating certain conditions. Other authors propose that the more common a family is in an area, the greater is the probability of its medicinal use [54].

Each group used different numbers of native and exotic species: Afrocaribbean (99 natives, 49 exotics); Amerindian (201 natives, 46 exotics); and Mestizo (63 natives, 44 exotics). The proportion of natives to exotics was significantly different in the Afrocaribbean (Z = 4.11, *P <* 0.01) and Amerindian (Z = 9.86, *P <* 0.01) communities, but not in the Mestizo communities (Z = 1.84, *P >* 0.01). There were significant differences in native and exotic plant use by groups (*X*^*2*^ = 21.95, *P <* 0.001). When compared between groups, significant differences in the proportion of natives to exotics were present between the Afrocaribbean and Amerindian communities (*X*^*2*^ = 10.63, *P <* 0.001), and between the Amerindian and Mestizo communities (*X*^*2*^ = 19.93, *P <* 0.001), but not between the Afrocaribbean and Mestizo communities (*X*^*2*^ = 1.72, *P =* 0.19). Therefore, native plant species represented a large portion of the medicinal plants used in the Amerindian (81%) and Afrocaribbean (67%) communities, and somewhat less in the Mestizo communities (59%). The high proportion of natives used by the Amerindians is both a result of millennia of cultural development in the region and a partial reflection of their preservation of indigenous knowledge in the face of historical events, i.e. a kind of cultural resistance. Hoffman [23] claims that indigenous groups that have lived in an area for long periods have a deeper knowledge of regional resources than non-indigenous groups that have lived there for less time. Incorporation of exotic plants into their Pharmacopoeia manifests the Amerindians’ flexibility in the adoption of new resources. This contributes to diversification of the medicinal species used by a group as an alternative health care option, which would agree with Albuquerque’s diversification hypothesis [55]. The overall Pharmacopoeia (natives and exotics plants) used by the Afrocaribbean and Mestizo groups is the result of the integration of mainly African, European and native components, and manifests the genetic and cultural mixture of regional historical processes. During approximately three hundred years, Africans arrived in the region as slaves. They brought their own therapeutic knowledge with them, but, due to the inherent restrictions of slavery, initially had access only to plant resources (mainly from Europe, Africa and Asia) directly introduced by Europeans as crops, ornamentals and medicinal [4,7,8,56]. Before indigenous populations were decimated in some areas, African immigrants were able to exchange knowledge about natural resource use with indigenous groups [57]. As Brussell observes [58], the rapid destruction of indigenous populations in some portions of the Basin did not necessarily lead to destruction of their natural resources and their knowledge of them. Afrocaribbean populations managed to incorporate a large number of native plant species into their therapeutic practices. They experimented with resources similar to those they had known in their points of origin, and very probably used organoleptic analysis and intuition, as has been the case in other human groups [6,59]. One example is the Camdomblé religious traditions of Salvador, Bahía, Brazil, which include both native and exotic *Ficus* species [60]. The less proportion of native plants used by the Mestizos is the result of a complex set of interactions between diverse factors mainly historical and cultural ones, since a short occupation of a territory limits the acquisition of extensive ethnobotanical knowledge which are orally transmitted. However, the influence of diverse cultures to which they have been exposed, have generated an open identity to accept resources from different sources, so a greater number of exotic species incorporated in their Pharmacopoeia is observed.

### Main diseases treated with medicinal plants by Afrocaribbeans, Amerindians and Mestizos

Among the three analyzed groups, the largest number of medicinal plant species used were for infectious and parasite diseases; symptoms and signs; digestive system diseases; and respiratory system diseases (Table 3). The greater diversity of plants used to treat these disorders in these communities may be due to their frequency and the risk that they represent in public health terms. In a 2004 report, the WHO listed twelve diseases responsible for the largest number of deaths among women and men worldwide, which included infectious and parasite diseases; respiratory system infections; respiratory system diseases; and digestive system diseases [61,62]. The WHO also listed the twenty diseases with the highest death rates for all ages, ranking infectious respiratory diseases in third place (4.2 million deaths) and diarrheal diseases in fifth place (2.2 million deaths) [61].

**Table 3:** **Number of medicinal species used in each category of disease for the three groups**

**Categories of disease**	**Afrocaribeans**	**Amerindians**	**Mestizos**
**Number of species**			
Infectious and parasitic diseases (diarrhea, intestinal parasites, thrush)	47	83	23
Symptoms, signs and abnormal findings (headache, fever, itching)	46	82	32
Diseases of the digestive system (stomach cramps, flatulence, gastritis)	41	82	40
Diseases of the respiratory system (asthma, flu, cough)	31	53	48
Diseases of the genitourinary system (urinary infections, kidney stones, inflammation of ovaries)	28	39	28
Injury, poisoning and certain other consequences of external causes (wound, poisoning, scorpion stings, snake bite)	24	23	4
Diseases of the skin and subcutaneous tissue (boils, abscesses, sores).	23	49	12
Diseases of the circulatory system (hypertension, heart palpitations, circulatory problems)	23	4	19
Diseases of the musculoskeletal system (fractures, back pain, inflammation blow)	21	24	6
Diseases of the nervous system (attacks of nerves, hysteria, epilepsy)	8	11	14
Diseases of the ear and mastoid process (earache)	4	12	3
Endocrinal, nutritional and metabolic diseases (anemia, uric acid, diabetes)	2	12	2
Pregnancy, childbirth and the puerperium (birth complications, retained placenta, lack of milk).	0	4	4
Diseases of the eye and adnexa (burning eyes, fleshy eye, eye irritation)	0	5	1

Listed between brackets are 3 examples of ailments we recorded in each category.

### Species use value in the three studied groups

Mean species UV values in each group did not differ (H = 2.09; 0.35 Kruskal-Wallis) with 0.88 ± 0.83 for the Afrocaribbean communities, 0.94 ± 1.01 for the Amerindian communities and 0.99 ± 0.81 for the Mestizo communities. The Afrocaribbean communities had 36% species with a use value greater than or equal to its mean use value, the Amerindian communities had 34% and the Mestizo communities had 42%.

Among the Afrocaribbean communities, the highest use value (5.33) was for *Momordica charantia* L., used to treat skin and subcutaneous diseases; endocrine, nutritional and metabolic diseases; infectious and parasite diseases; symptoms and signs; cardiovascular system diseases; digestive system diseases; genital-urinary system diseases; and muscle-skeletal system diseases. For the Amerindian communities, the highest use value (7.33) was for *Aloe vera* (L.) Burm, used to treat skin and subcutaneous diseases; endocrine, nutritional and metabolic diseases; injuries, poisoning and other external consequences; cardiovascular system diseases; and digestive system diseases. Among the Mestizo communities, the highest use value (4.00) was for *Plectranthus amboinicus* (Lour.) Spreng, used in treatment of ear diseases; symptoms and signs; cardiovascular system diseases; digestive system diseases; nervous system diseases; and respiratory system diseases (Table 4).

**Table 4:** **Species with use value ≥ mean species UV values in each group**

**Species and families**	**Categories ICD-WHO**	**Afro**	**Ame**	**Mes**	**R**	**Voucher number**
*Momordica charantia* L. - **Cucurbitaceae**	I, IV, VI, IX, X, XI, XII, XIII, XIV, XVIII, XIX	5.33	1.33	3.33	E	García 2329
*Citrus aurantium* L. - **Rutaceae**	I, VI, X, XI, XIII, XVIII, XIX	4.67	4.00	1.67	E	Jiménez 1507
*Citrus aurantiifolia* (Chrisym.) Swimgle. **Rutaceae**	I, IV, VI, IX, X, XI, XIV, XVIII, XIX	3.33	6.67	1.67	E	Jiménez 1499
*Chromolaena odorata* (L.) R.M. King & H. Rob. **Asteraceae**	I, VI, X, XI, XII, XIV, XVIII, XIX,	2.67	1.33	1.00	N	Medina 181
*Aloe vera* (L.) Burm. - **Xanthorrhoeaceae**	I, IV, VI, IX, XI, XII, XIII, XIV, XVIII, XIX	2.00	7.33	3.00	E	Jiménez 1525
*Petiveria alliacea* L.- **Phytolaccaceae**	X, XI, XII, XIII, XVIII	1.67	2.33	1.33	N	Jiménez 24
*Annona muricata* L. - **Annonaceae**	I, VI, IX, X, XI, XVIII	1.33	3.33	2.67	N	FORPLAN 1695
*Psidium guajava* L. - **Myrtaceae**	I, X, XI, XII, XIII, XVIII, XIX	1.33	3.33	1.67	N	Jiménez 41
*Persea americana* Mill. - **Lauraceae**	I, IV, X, XII, XIII, XIV, XVIII	1.00	4.33	1.67	N	Girón 245
*Chenopodium ambrosioides* L. - **Amaranthaceae**	I, IX, XI, XVIII	1.00	2.33	1.67	N	Jiménez 1511
*Ricinus communis* L.- **Euphorbiaceae**	I, X, VIII, XI, XII, XIII, XIV, XVIII, XIX	3.33	1.67	0	E	Jiménez 47
*Citrus sinensis* (L.) Osbeck - **Rutaceae**	I, X, XI, XIII, XVIII	3.33	0	0	E	Veloz 3010
*Gossypium barbadense* L. - **Malvaceae**	I, VIII, XI, XII, XIII, XIV, XVIII, XIX	3.00	0	0	E	García 2588
*Saccharum officinarum* L. - **Poaceae**	I, VI, XI, XII, XIX	3.00	0	0	E	Mejía 9024
*Lantana camara* L. - **Verbenaceae**	X, XI, XVIII	2.33	0	0	N	Girón 197
*Zea mays* L. -**Poaceae**	I, IX, XII, XIII, XIV, XVIII, XIX	2.00	1.33	0	N	Girón 240
*Musa paradisiaca* L. - **Musaceae**	I, XIII, XVIII	2.00	0	1.00	E	Jiménez 691
*Kalanchoe pinnata* (Lam.) Pers. - **Crassulaceae**	IX, X, XIII	2.00	0	0	E	Ochoa 274
*Haematoxylum campechianum* L. - **Fabaceae**	I, XIX, XVIII, XI	2.00	0	0	N	Rouzier 104
*Ocimum gratissimum* L. - **Lamiaceae**	I, XI	2.00	0	0	E	Mejía 1399
*Ocimum campechianum* Mill. - **Lamiaceae**	I, VIII, XI, XII, XVIII, XIX	1.67	3.67	0	N	Medina 276
*Allium sativum* L. - **Amaryllidaceae**	I, IX, X, XI, XII, XV	1.67	3.33	0	E	Jiménez 1519
*Guazuma ulmifolia* Lam. - **Malvaceae**	I, IX, X, XIII	1.67	0	0	N	Pimentel 1164
*Zingiber officinale* Roscoe - **Zingiberaceae**	I, X, XI, XIII, XIV, XVIII	1.33	2.33	0	E	Ochoa 315
*Carica papaya* L. - **Caricaceae**	I, VIII, IX, XI, XIII	1.33	1.67	0	N	Girón 227
*Mangifera indica* L. - **Anacardiaceae**	I, IX, XII, XIV	1.33	0	0	E	Girón 810
*Spondias mombin* L. - **Anacardiaceae**	I, XII, XIII, XIV	1.33	0	0	N	Medina 84
*Crescentia cujete* L. - **Bignoniaceae**	VI, XII, XIII, XIV	1.33	0	0	N	Jiménez 22
*Terminalia catappa* L. - **Combretaceae**	IX, XI	1.33	0	0	E	Arvigo 1061
*Cucurbita moschata* Duchesne - **Cucurbitaceae**	I, XIV, XVIII	1.33	0	0	N	Jiménez 127
*Abelmoschus esculentus* (L.) Moench - **Malvaceae**	I, XIV, XVIII, XIX	1.33	0	0	E	Jiménez 683
*Moringa oleífera* Lam. - **Moringaceae**	X, XI, XVIII	1.33	0	0	E	Rouzier 129
*Ocimum basilicum* L. - **Lamiaceae**	I,VI, VII, IX, X, XVIII	1.00	3.67	0	E	Girón 168
*Nicotiana tabacum* L. - **Solanaceae**	VIII, XII, XIX, XVIII, XIX	1.00	1.67	0	N	Girón 130
*Tournefortia hirsutissima* L. - **Boraginaceae**	X, XVIII	1.00	1.67	0	N	Veloz 3024
*Lippia graveolens* Kunth - **Verbenaceae**	VIII, XIV, X, XI, XVIII	0	3.67	0	N	Ocampo 88
*Bursera simaruba* (L.) Sarg. - **Burseraceae**	I, XI, XII, XVIII, XIX	0	3.33	1.00	N	Aker 492
*Hamelia patens* Jacq. - **Rubiaceae**	VII, VIII, XII, XVIII, XIX	0	3.33	0	N	Medina 173
*Ruta chalepensis* L. - **Rutaceae**	I, VI, VIII, X, XI, XIV, XVIII	0	3.33	0	E	Medina 236
*Parthenium hysterophorus* L. - **Asteraceae**	X, XII, XIII, XIV, XIX	0	3.00	0	N	Arvigo 1097
*Cymbopogon citratus* (DC.) Stapf - **Poaceae**	IX, X, XI, XIV, XVIII	0	2.67	1.33	E	García 2654
*Cecropia peltata* L. - **Urticaceae**	I, IV, X, XI, XIII, XIV	0	2.67	1.00	N	Medina 215
*Allium schoenoprasum* L. - **Amaryllidaceae**	I, VIII, X, XI	0	2.67	0	E	Medina 125
*Spondias purpurea* L. - **Anacardiaceae**	I, XI, XII, XVIII	0	2.67	0	N	Medina 84
*Cocos nucifera* L. - **Arecaceae**	I, XI, XII, XIV, XVIII	0	2.33	1.33	E	Jiménez 1512
*Piper auritum* Kunth - **Piperaceae**	X, XI, XII, XIII, XV, XVIII	0	2.33	1.33	N	Girón 273
*Capraria biflora* L. - **Schrophulariaceae**	I, XII, XIV	0	2.33	1.00	N	Medina 27
*Artemisia ludoviciana* Nutt. - **Asteraceae**	I, X, XI	0	2.33	0	E	Medina 53
*Tagetes erecta* L. - **Asteraceae**	X, XI, XII, XIII, XVIII	0	2.33	0	N	Medina 237
*Bixa orellana* L. - **Bixaceae**	I, XI, XVIII	0	2.33	0	N	Medina 267
*Struthanthus orbicularis* (Kunth) Blume **Lorantaceae**	XI, XII, XIII, XIV	0	2.33	0	N	Medina 224
*Byrsonima crassifolia* (L.) Kunth - **Malpighiaceae**	I, XII, XVIII	0	2.33	0	N	Medina 69
*Eryngium foetidum* L. - **Apiaceae**	I, X, XI, XVIII	0	2.00	0	N	Jiménez 125
*Kalanchoe integra* (Medik.) Kuntze **Crassulaceae**	I, X, XII, XVIII	0	2.00	0	E	Medina 8
*Plantago major* L. - **Plantaginaceae**	I, XI, XII, XIV, XVIII	0	1.67	2.00	E	Medina 48
*Heliotropium angiospermum* Murray **Boraginaceae**	I, X, XI, XIX	0	1.67	0	N	Medina 152
*Clinopodium ludens* (Shinners) A Pool. **Lamiaceae**	I, X, XI	0	1.67	0	N	Medina 50
*Brosimum alicastrum* Sw. - **Moraceae**	VI, X, XV, XIX	0	1.67	0	N	Paredes 254
*Manilkara zapota* (L.) P. Royen - **Sapotaceae**	I, IV	0	1.67	0	N	Medina 39
*Plectranthus amboinicus* (Lour.) Spreng. **Lamiaceae**	VI, VIII, IX, X, XI, XVIII	0	1.33	4.00	E	García 7541
*Annona squamosa* L. - **Annonaceae**	I, IX, X, XVIII	0	1.33	2.00	N	Medina 55
*Anacardium occidentale* L. -**Anacardiaceae**	I, X	0	1.33	1.00	N	FLORPAN 1870
*Rauvolfia tetraphylla* L. -**Apocynaceae**	I, VII, XII	0	1.33	0	N	Méndez 2476
*Aristolochia maxima* Jacq. - **Aristolochiaceae**	IV, XI, XIX	0	1.33	0	N	Medina 171
*Aristolochia odoratissima* L.- **Aristolochiaceae**	X, XVIII	0	1.33	0	N	FLORPAN 5695
*Ehretia tinifolia* L. - **Boraginaceae**	X, XV, XVIII	0	1.33	0	N	Méndez 2448
*Drymaria cordata* (L.) Willd. ex Schult. **Caryophyllaceae**	X, XI, XVIII	0	1.33	0	N	Jiménez 1353
*Mentha citrata* Ehrh - **Lamiaceae**	I, XI	0	1.33	0	E	Medina 287
*Pilocarpus racemosus* Vahl - **Rutaceae**	XI, XIII, XVIII	0	1.33	0	N	Medina 203
*Urera baccifera* (L.) Gaudich. ex Wedd. **Urticaceae**	XIII, XVIII, XIX	0	1.33	0	N	Medina 289
*Mentha nemorosa* Willd. - **Lamiaceae**	I, IX, X, XI, XVIII	0	0	2.67	E	Roig 4621
*Justicia pectoralis* Jacq. - **Acanthaceae**	VI, IX, XI, XVIII	0	0	2.33	N	Martinez 4758
*Pluchea carolinensis* (Jacq.) G. Don. **Asteraceae**	VI, IX, XVIII	0	0	2.33	N	Medina 159
*Mentha piperita* L. - **Lamiaceae**	VI, IX, X, XI, XVIII	0	0	2.33	E	Medina 54
*Origanum majorana* L. - **Lamiaceae**	I, X, XI, XVIII	0	0	2.33	E	Boucourt 4759
*Ruta graveolens* L.- **Rutaceae**	VI, VIII, X, XI, XVIII	0	0	2.33	E	Soberats 90-04
*Bidens pilosa* L. - **Asteraceae**	X, XI, XII, XVIII	0	0	2.00	N	Boucourt 4767
*Ocimum sanctum* L. - **Lamiaceae**	I, IV, IX, X, XI, XVIII	0	0	2.00	E	Mejía 9143
*Simarouba glauca* DC. - **Simaroubaceae**	VI, X, XVIII	0	0	2.00	N	Jiménez 40
*Lippia alba* (Mill.) N.E. Br. - **Verbenaceae**	VI, VIII, XI, XVIII	0	0	2.00	N	FLORPAN 1933
*Turnera ulmifolia* L. **Passifloraceae**	I, VI, XI, XIV	0	0	1.67	N	Méndez 149
*Tradescantia spathacea* Sw. - **Commelinaceae**	X, XIII, XV	0	0	1.33	N	Jiménez 30
*Rhizophora mangle* L. -**Rhizophoraceae**	XI, XII, XIV	0	0	1.33	N	Durán 433

Categories ICD-WHO: I = Infectious and parasitic diseases; IV = Endocrinal, nutritional and metabolic diseases; VI = Diseases of the nervous system; VII = Diseases of the eye and adnexa; VIII = Diseases of the ear and mastoid process; IX = Diseases of the circulatory system; X = Diseases of the respiratory system; XI = Diseases of the digestive system; XII = Diseases of the skin and subcutaneous tissue; XIII = Diseases of the musculoskeletal system; XIV = Diseases of the genitourinary system; XV = Pregnancy, childbirth and the puerperium; XVIII = Symptoms, signs and abnormal findings; XIX = Injury, poisoning and certain other consequences of external causes.

Afro = Afrocaribbeans; Ame = Amerindians ; Mes: Mestizos; R = Natural range (N = Native plants; E = Exotic plants).

Some species had high use value (i.e. greater than or equal to the mean) in all three groups: *Aloe vera*; *Citrus aurantiifolia* (Chrisym.) Swimgle; *Citrus aurantium* L.; *Chenopodium ambrosioides* L.; and *Psidium guajava* L. In contrast, other high use value species were only such in one of the groups: Afrocaribbean, *Gossypium barbadense* L., *Saccharum officinarum* L., *Haematoxylum campechianum* L., *Ocimum gratissimum* L. and *Spondias mombin* L.; Amerindian, *Lippia graveolens* Kunth, *Ruta chalepensis* L., *Punica granatum* L., *Byrsonima crassifolia* (L.) Kunth, and *Struthanthus orbicularis* (Kunth) Blume; and Mestizo, *Mentha nemorosa* Willd., *Origanum majorana* L. *Ruta graveolens* L., *Justicia pectoralis* Jacq. and *Bidens pilosa* L.

Although 60% of the high use value species in all three groups are natives, the two species with the highest use value in all three groups are exotics. This may result from the interviewees being almost all housewives with greater access to cultivated species in family gardens or crop systems. In addition, these species have other uses, such as food. Bennett and Prance [6] report similar findings in South America where use of exotics is common because medicinal value is assigned to plants used primarily as food; for example, *Citrus aurantium, Citrus aurantifolia, Cocos nucifera* and *Allium cepa*. Exotic species are normally cultivated and can be found in family gardens, making them easily available.

### Similarity and differences in the use of medicinal plant species between groups

All communities compared in pairs showed similarity index values around 27%. The Afrocaribbean and Amerindian communities had 54 plant species in common, the Afrocaribbean and Mestizo communities had 35 in common and the Amerindian and Mestizo communities had 48 in common. This similarity may be due to migrations within the Caribbean Basin and consequent cultural intermixing. Segregation of the medicinal plant species repertoires used by the three groups can be explained by the different origin and respective cultural prevalence of each ethnic community, which would result in species exclusive to each group. Several studies state that human migration influences local medicinal knowledge as new remedies are introduced [63-65]. The present results demonstrate this enrichment of medicinal knowledge in that the Amerindian, Afrocaribbean and Mestizo groups clearly adapted their medicinal plant repertoires to incorporate both native and exotic species and also the medicinal plant diversity each group knows, since they only share 27% of species between groups.

**Figure 1:**
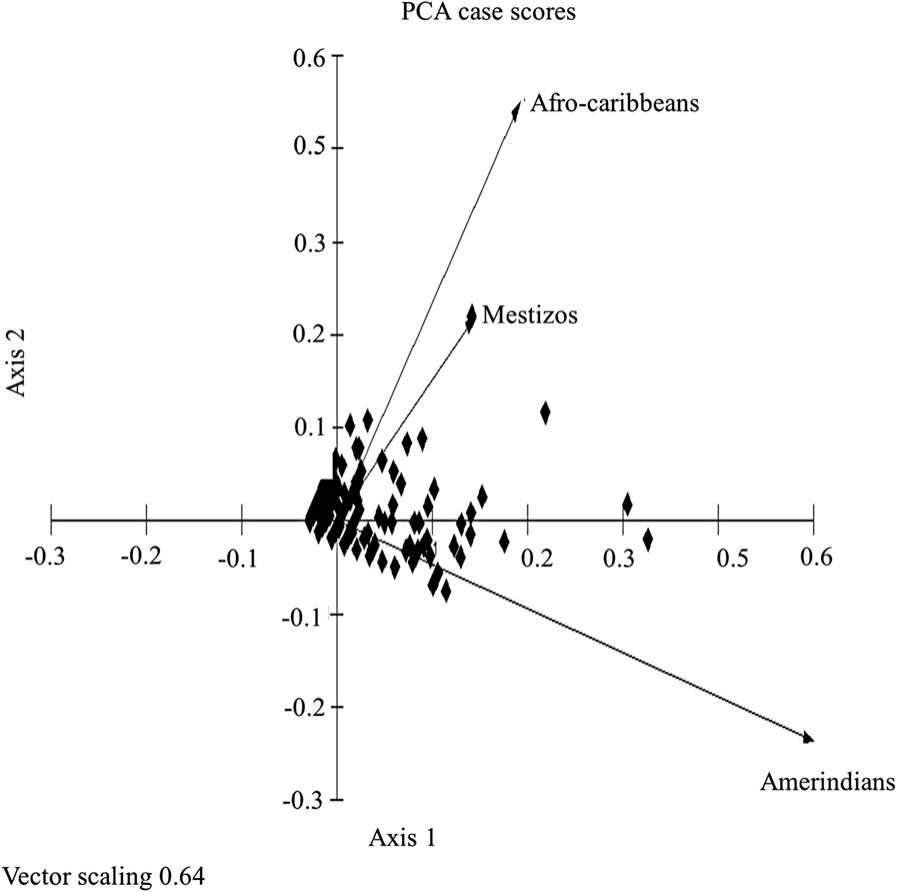
**Principle Component Analysis (PCA) with projection of the variables (Afrocaribbean, Amerindians and Mestizos) and the observations (use values of medicinal species) in the plane of axes 1 and 2.**

In the PCA analysis considering plant species use value in each group, the principal axis explained 57% of variation and clearly separated the Amerindian group from the Afrocaribbean and Mestizo groups (0.901 correlation) (Figure 1). Axis two explained 24% of variation and separated the Afrocaribbean group from the Amerindian and Mestizo groups (0.812 correlation). The Mestizo group exhibited very low correlation between the axes. This analysis reflects differences between the groups based on species use value. The point cluster near the union of the three vectors corresponds to species with similar values in two and even all three groups, whereas the points along the vectors are high use value species used exclusively by one of the groups (Figure 1).

Eighty-three species were exclusive used by the Afrocaribbean group, of which 57 are native and 26 exotic; of these, high use value species include *Saccharum officinarum* (3.00), *Gossypium barbadense* (3.00), *Haematoxylum campechianum* (2.00), *Ocimum gratissimum* (2.00), *Abelmoscus esculentus* (L.) Moench (1.33), *Spondias mombin* L. (1.33), and *Moringa oleifera* Lam. (1.33), among others. Species with high use value in this group, but that were not exclusive to it include *Momordica charantia* (5.33), *Ricinus communis* L., (3.33) *Citrus sinensis* (L.) Osbeck (3.33), *Chromolaena odorata* (L.) R.M. King & H. Rob. (2.67), *Lantana camara* L. (2.33), and *Annona muricata* L. (2.33), among others.

One hundred sixty-nine species were exclusive to the Amerindian group, of which 150 were natives and 19 exotic. Exclusive species with the highest UV included *Lippia graveolens* (3.67), *Ruta chalepensis* (3.33), *Punica granatum* (3.33), *Artemisia ludoviciana* Nutt. (2.33), *Byrsonima crassifolia* (2.33), *Bixa orellana* L. (2.33), *Tagetes erecta* L. (2.33), and *Struthanthus orbicularis* (Kunth) Blume (2.33), among others. High use value species shared with other groups included *Aloe vera* (7.33), *Persea americana* Mill. (4.33), *Ocimum basilicum* L. (3.67), *Lippia graveolens* (3.67), and *Ocimum campechianum* Mill. (3.67), among others.

In the Mestizo group, 48 species were exclusive, 26 of which were native and 22 exotic. High use value exclusive species in this group included *Mentha nemorosa* (2.67), *Origanum majorana* L. (2.33), *Ruta graveolens* (2.33), *Justicia pectoralis* (2.33), *Bidens pilosa* (2.00), and *Simaruba glauca* D.C. (2.00), among others. Shared species with high use value included *Plectranthus amboinicus* (4.00), *Mentha nemorosa* (2.67), *Pluchea carolinensis* (Jacq.) G. Don (2.33) and *Tamarindus indica* L. (2.33), among others.

Mixture of native and exotic species by immigrant groups is also a manifestation of their efforts to survive in new environments by mixing indigenous knowledge with their own knowledge. This is a kind of knowledge hybridization like that described by Coe and Anderson [30] among Afrocaribbean groups influenced by the Miskito, Suma and Rama indigenous groups in Nicaragua. Another example is the medicinal plant repertoire used on the island of Montserrat, of which only 15% is of Amerindian origin [66]. Medeiros and his collaborators [67] suggest five factors that affect incorporation of local pharmacopeia resources and knowledge, among them the prevailing health conditions in populations and the intensity of contact between immigrant and local populations. When considered in tandem with the floral richness of the Caribbean Basin derived from its biogeographic history, these two factors help to better understand the amalgam of plant species used for therapeutic purposes among the Basin’s populations. Even so, the influences of migration, cultural mixture and knowledge hybridization in the region have not blurred the clear differences between the studied groups’ repertoires of medicinal plants used for primary health care.

## Conclusions

Although migration, cultural intermixing and a consequent hybridization of medicinal plant knowledge have occurred in the Caribbean Basin, the results highlight differences between the three studied groups in terms of the medicinal plant repertoire they employ for primary health care.
